# Case Report: Dual molecular diagnosis in complex congenital heart disease in an Ecuadorian patient with supravalvar aortic stenosis and pulmonary valve stenosis carrying pathogenic variants in *ELN* and *BRAF* gene

**DOI:** 10.3389/fped.2026.1868988

**Published:** 2026-07-13

**Authors:** Santiago Cadena-Ullauri, Viviana A. Ruiz-Pozo, Rafael Tamayo-Trujillo, Patricia Guevara-Ramírez, Elius Paz-Cruz, Rodrigo Bossano R, Miguel Hinojosa, Paul Onofre-Ruiz, Ana Karina Zambrano

**Affiliations:** 1 Universidad UTE, Facultad de Ciencias de la Salud Eugenio Espejo, Centro de Investigación Genética y Genómica, Quito, Ecuador; 2 Private Practice Pediatric Cardiologist, Quito, Ecuador; 3 Universidad UTE, Facultad de Ciencias de la Salud Eugenio Espejo, Quito, Ecuador

**Keywords:** cardiovascular disease, case report, genetics, genomics, healthcare

## Abstract

**Background:**

Congenital heart diseases (CHDs) are the most common inherited anomalies worldwide and remain a major cause of mortality in pediatric populations. Advances in genomic medicine have improved the ability to identify molecular mechanisms underlying complex CHD phenotypes, including cases not fully explained by a single-gene disorder.

**Case presentation:**

This case presents an Ecuadorian girl with the coexistence of congenital supravalvar aortic stenosis and congenital valvar pulmonary stenosis. Molecular analysis identified pathogenic variants in *ELN* and *BRAF*, together with a *NOTCH1* p.(Leu2429Arg) variant of uncertain significance (VUS). Ancestry analysis revealed an admixed background with predominant European ancestry, followed by Native American and African components.

**Discussion:**

Findings are consistent with a multilocus in which ELN and BRAF variants may biologically plausibly contribute to the observed phenotype; however, this should be taken cautiously as more evidence, and studies must be performed to understand the impact of these variants. This case also highlights the importance of generating cardiogenetic evidence from underrepresented Latin American populations.

**Conclusion:**

Comprehensive genomic evaluation can refine etiologic diagnosis, reveal blended mechanisms in CHD, and support precision medicine approaches. This report contributes to the growing role of advanced genetic testing in the future management of CHD.

## Introduction

Congenital heart diseases (CHDs) are the most common congenital malformations worldwide, affecting approximately 0.8% to 1.2% of live births, and represent a major cause of morbidity and mortality related to congenital anomalies in infants and children (1–4). CHDs comprise a heterogeneous group of structural abnormalities involving the heart and/or great vessels, and its clinical severity varies widely, ranging from conditions that may not require clinical intervention to serious malformations requiring multiple clinical interventions and the need of lifelong management (5–11). Therefore, early diagnosis and individualized management are essential to reduce complications, optimize therapeutic decision-making, and improve long-term outcomes (3, 4, 10).

According to the International Paediatric and Congenital Cardiac Code (IPCCC) and the ICD-11 congenital cardiology nomenclature, CHDs are classified within the level 0 category of “Structural developmental anomaly of heart and great vessels.” In this hierarchical framework, the lesions described in the present case correspond to “Congenital anomaly of a ventriculo-arterial valve or adjacent regions” in level I, which includes congenital pulmonary valvar stenosis and congenital supravalvar aortic stenosis in level III (12).

Congenital supravalvar aortic stenosis is a rare form of left ventricular outflow tract obstruction characterized by narrowing of the sinotubular junction or ascending aorta (13–15). This condition may occur as an isolated lesion, as a familial disorder, or in association with Williams syndrome (15). Pathogenic alterations resulting in haploinsufficiency of the *ELN* gene, which encodes elastin, are strongly associated with this condition (15–18).

Congenital pulmonary valvar stenosis is a congenital cardiovascular malformation of the pulmonary valve in which there is narrowing or obstruction to flow from the right ventricle to the pulmonary circulation (19–21). This lesion is also a recognized feature of several developmental syndromes, particularly the RASopathies (22, 23). Among these disorders, germline pathogenic variants in the *BRAF* gene have been associated with cardiovascular manifestations, including congenital pulmonary valvar stenosis (24–26).

Although advances in prenatal screening, echocardiography, surgical correction, and intensive care have significantly improved survival, the etiological diagnosis of CHD remains incomplete in many patients (11, 27–30). In this context, genetic alterations play a central role in the development of CHD, particularly in complex, syndromic, familial, or early-onset presentations (31, 32). Therefore, genetic evaluation has become increasingly relevant not only for understanding disease pathogenesis, but also for refining clinical diagnosis and prognosis (10, 33).

The clinical impact of genetic testing in CHD extends beyond variant detection, particularly with the implementation of Next-Generation Sequencing (NGS), which has improved the identification of syndromic conditions, monogenic causes, and cases involving multiple clinically relevant variants (31, 32, 34). Identifying pathogenic or likely pathogenic variants can help distinguish isolated from syndromic forms, explain extracardiac manifestations, guide surveillance, support risk stratification, and inform recurrence-risk estimation, reproductive counseling, and cascade testing (31, 32). At the population and research level, NGS has contributed to understanding molecular pathways involved in cardiac development; however, in individual case reports, these findings should be interpreted as supporting genotype–phenotype assessment rather than as definitive evidence of disease mechanism (10, 32, 35–38).

Furthermore, ancestry-informed patient management can also provide an additional layer for personalized management, as population-specific genetic backgrounds can influence susceptibility, clinical expression, and diagnostic yield in CHD. This perspective is particularly relevant in underrepresented and resource-limited settings, where access to advanced molecular diagnostics remains restricted and local genomic data are still scarce (10, 39–44).

In the present case report, we describe a child with the coexistence of congenital supravalvar aortic stenosis and congenital valvar pulmonary stenosis whose molecular evaluation identified pathogenic variants in *ELN* and *BRAF*. This case highlights the value of genomic testing for identifying clinically relevant variants that may help contextualize complex CHD phenotypes, refine diagnostic interpretation, and support more individualized patient care, while recognizing that causality cannot be established from a single case alone.

## Case description

**Figure 1:**
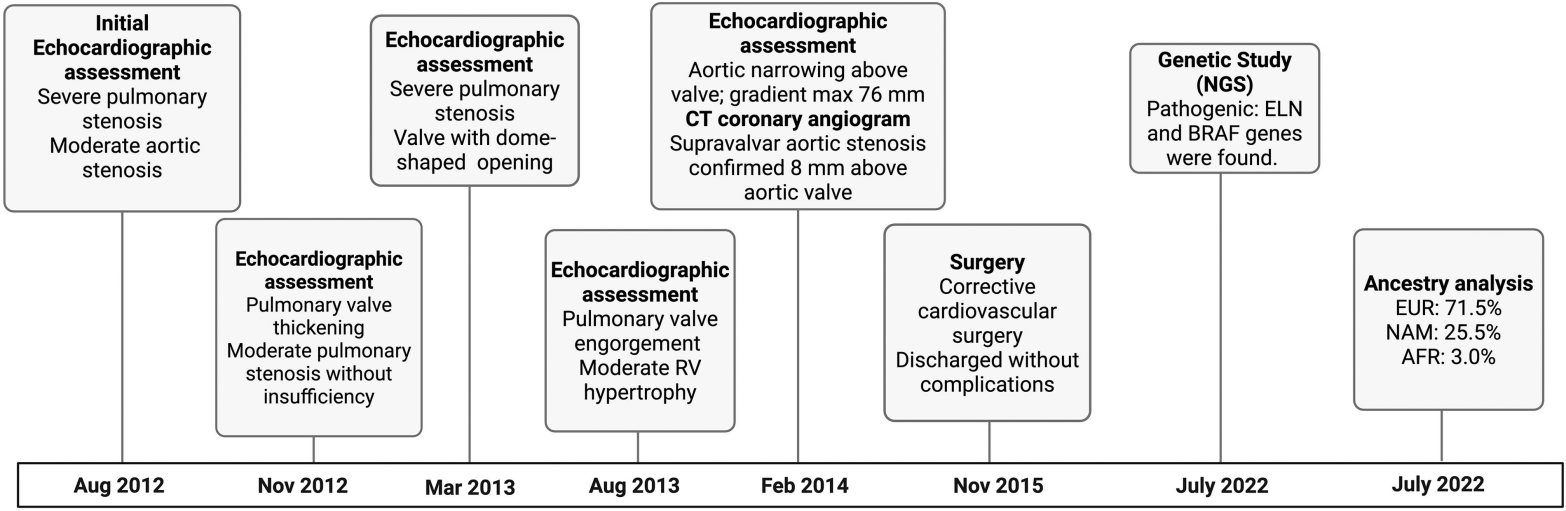
Chronological timeline of the patient's clinical course. Created in BioRender. Cadena-Ullauri S., (2026) https://BioRender.com/9eraq67.

This case report describes an Ecuadorian female pediatric patient whose medical history revealed two congenital cardiovascular anomalies: pulmonary valvar stenosis and supravalvar aortic stenosis. The patient was first evaluated for cardiovascular disease at 1 year of age, underwent surgical correction at 4 years of age, and genomic analysis was performed at 11 years of age. On physical examination, craniofacial features were identified, including hypertelorism, broad forehead, coarse facial features, and prognathism. However, aside from the craniofacial features identified during physical examination, the available clinical evaluations did not document any additional neurodevelopmental findings, extracardiac anomalies, chronic comorbidities, drug allergies, or relevant family history. In addition, a complete vaccination history was confirmed in strict accordance with the national immunization schedule. A detailed timeline summarizes the episodes of care (Figure 1).

### Echocardiographic findings

Longitudinal echocardiographic evaluations were performed over a three-year period to monitor the progression of the identified lesions. The initial detailed assessment, conducted in August 2012 at one year of age, confirmed situs solitus and levocardia. Moderate right ventricular (RV) hypertrophy and a thickened pulmonary valve with a characteristic dome-shaped opening were evidenced. These findings were consistent with severe pulmonary stenosis and an additional component of infundibular obstruction. Significant hypertension was observed within the right ventricle as a result of the outflow tract obstruction, although left ventricular morphology and systolic function remained preserved.

Subsequent follow-up evaluations demonstrated a variable hemodynamic profile. In November 2012, a control echocardiogram documented a transition of pulmonary stenosis to a moderate grade, although pulmonary hypertension persisted with a recorded systolic pressure of 60 mmHg.

Furthermore, evaluations conducted between March and August 2013 identified a slightly dilated pulmonary ring, measuring between 1.4 cm and 1.5 cm. During this interval, the patient's condition showed further variability, eventually progressing back to severe stenosis accompanied by mild valvular insufficiency. Throughout the monitoring period, coronary anatomy remained normal and the remaining transvalvular flows were found to be physiological.

A CT Coronary Angiogram performed on February 26, 2014, confirmed supravalvar aortic stenosis. The lesion was projected in the ascending aorta, approximately 8 mm above the level of the aortic valve. The defect measured 8 mm in length and produced a focal reduction in vascular caliber: the diameter at the level of the stenosis was 7.5 mm, compared to 11 mm immediately above and below the lesion.

No interatrial or intra-atrial defects were identified. The pulmonary artery and its main branches showed no alterations, and pulmonary veins and venae cavae appeared normal. Cardiac size was normal. Coronary arteries were not adequately assessed as the study was performed at the patient's baseline heart rate (Table 1).

**Table 1:** Summary of serial echocardiographic findings (2012–2014).

Date	EF (%)	Right ventricular wall thickness (mm)	Peak velocity (m/s)/Pulmonary artery pressure gradient (mmHg)	Pulmonary artery systolic pressure (mmHg)	Indexed aortic valve area (cm^2^/m^2^)	Key finding
Aug 2012	77	1.1	3.83/59	60	0.80	Severe pulmonary stenosis; moderate aortic stenosis
Nov 2012	75	1.1	3.87/60	60	0.92	Pulmonary valve engorgement; severe aortic stenosis without insufficiency
Mar 2013	77	1.3	3.88/61	50	1.08	Severe pulmonary stenosis; valve with dome-shaped opening.
Aug 2013	74	1.4	3.90/63	50	0.88	Pulmonary valve engorgement; severe stenosis; moderate RV hypertrophy
Feb 2014	67	1,6	4.03/65	50	0.95	Severe pulmonary stenosis, severe aortic stenosis, RV hypertrophy

EF, ejection fraction; RV, right ventricular.

Intraoperative findings confirmed severe congenital supravalvar aortic stenosis associated with a mild pulmonary gradient and significantly thickened aortic walls. Surgical reconstruction involved aortic root enlargement utilizing three autologous pericardial patches for tri-sinus augmentation, with an additional patch placed in the ascending aorta. The postoperative course was uneventful. At the time of discharge, the patient demonstrated a stable clinical condition (NYHA Functional Class I) and was maintained on a pharmacological regimen of captopril (6.25 mg) and furosemide (10 mg) daily.

### Genomic analyses

Before the collection and processing of the biological sample, written informed consent was obtained from the patient's legal guardians. Genomic DNA was extracted from peripheral blood collected in EDTA tubes using the PureLink Genomic DNA Mini Kit (Life Technologies). DNA integrity was assessed using the NanoDrop 2000 spectrophotometer and Qubit 4 fluorometer (Thermo Scientific). For the genomic assay, the TruSight Cardio panel (Illumina), which covers 174 genes associated with inherited cardiac conditions, was utilized on the MiSeq System (Illumina). Bioinformatics analysis was performed using Dragen Enrichment v.4.2.4. Variant classification was conducted through the Franklin platform and Variant Interpreter (Illumina).

Next-generation sequencing (NGS) analysis identified a heterozygous pathogenic variant in the *ELN* gene (c.1621C>T; p.Arg541Ter), which introduces a premature stop codon and is classified as Pathogenic according to ACMG/AMP guidelines. This mutation is predicted to result in elastin haploinsufficiency, which is consistent with the observed congenital supravalvar aortic stenosis phenotype. Furthermore, the genomic profile revealed two additional heterozygous variants, a pathogenic *BRAF* variant (c.1460T>G; p.Val487Gly), which could be related to abnormalities of the pulmonary valve, and a *NOTCH1* variant (7286T>G; p.Leu2429Arg), classified as a variant of uncertain significance (VUS) Table 2.

**Table 2:** Summary of identified genetic variants in the proband.

Gene	Genomic location (GRCh38)	HGVSC	HGVSP	Consequence	Zygosity	ACMG/AMP classification
*ELN*	7:74060184:T	c.1621C>T	p.(Arg541Ter)	stop_gained, splice_region_variant	Heterozygous	Pathogenic
*BRAF*	7:140778048:C	c.1460T>G	p.(Val487Gly)	missense_variant	Heterozygous	Pathogenic
*NOTCH1*	9:136496453:C	c.7286T>G	p.(Leu2429Arg)	missense_variant	Heterozygous	VUS*

* VUS: Variant of uncertain significance.

### Ancestry analyses

Genetic ancestry proportions were estimated using 46 ancestry-informative INDEL markers in a multiplex PCR reaction. Fragments were analyzed on a 3,500 Genetic Analyzer (Thermo Fisher Scientific). Data were processed using Data Collection v.3.3 and Gene Mapper v.5 software, and ancestry inference was performed with STRUCTURE v.2.3.4.

The individual's estimated ancestry composition revealed a predominant European component (71.5%), followed by a substantial Native American contribution (25.5%), and a minor African component (3.0%).

## Discussion

The present case describes a child with the uncommon coexistence of congenital supravalvar aortic stenosis and congenital pulmonary valvar stenosis, together with pathogenic variants in *ELN* and *BRAF*, and an additional *NOTCH1* variant classified as a VUS. This report is clinically relevant because the coexistence of two obstructive congenital cardiovascular lesions and multiple variants in genes involved in cardiovascular development raises the possibility of a multilocus genetic context. However, given the single-case design and the absence of functional and segregation studies, this interpretation should be considered hypothesis-generating rather than confirmatory.

The genetic architecture of CHD is increasingly being recognized as complex and multifactorial (45, 46). Large-scale genomic studies have shown that CHD can result from a broad spectrum of genetic contributions, including chromosomal abnormalities, copy-number variants, rare monogenic variants, *de novo* variants, inherited rare variants, oligogenic or multilocus combinations, noncoding regulatory variation, and polygenic background effects (46–48). These genetic factors may interact with environmental exposures, including extrinsic factors such as teratogens, alcohol, and hypoxia, as well as intrinsic maternal factors such as diabetes or obesity, and with detrimental events during embryogenesis (49).

In this context, oligogenic or multilocus models have gained increasing attention in CHD genetics, particularly because many cases cannot be fully explained by a single causal variant (50–52). This concept is also relevant to RASopathies, which are genetically heterogeneous disorders caused by dysregulation of the RAS/MAPK pathway and are characterized by variable expressivity and frequent cardiovascular involvement, including pulmonary valvar stenosis (24, 25, 53).). Therefore, the identification of pathogenic variants in *ELN* and *BRAF*, together with a *NOTCH1* VUS, may be consistent with the broader concept of multilocus complexity in CHD.

In the absence of segregation and functional data, the relative contribution of each variant cannot be weighted; therefore, it remains possible that one variant is primarily responsible for the phenotype, that another represents an incidental finding, or that the observed combination reflects additive, epistatic, or coincidental effects. Furthermore, this single case does not resolve the causal architecture of the phenotype and does not prove oligogenic inheritance, a blended phenotype, or gene–gene interaction. It should be interpreted as a descriptive study that supports cautious genotype–phenotype assessment and highlights the need for segregation studies, functional validation, and additional comparable cases.

Genetic variants in the *ELN* gene have been associated with congenital supravalvar aortic stenosis both as part of Williams-Beuren syndrome, usually caused by microdeletions, and as a non-syndromic form in which point mutations can cause congenital supravalvar aortic stenosis in an autosomal dominant pattern with incomplete penetrance (16, 18, 54–56). The *ELN* gene encodes elastin, a structural protein that confers elasticity to the extracellular matrix of various tissues and organs, including aortic smooth muscle (15, 16, 54). As a key component of the arterial wall, elastin is essential for maintaining vascular compliance and regulating blood flow and pressure (57). *ELN* haploinsufficiency consequently can potentially lead to insufficient elastin production, predisposing large arteries, including the aorta, to stenotic remodeling (16, 18, 54).

The elastin protein is composed of 786 amino acids (58, 59). The variant identified in this case is a nonsense mutation that a premature stop codon (PTC) at amino acid position 541, potentially affecting protein function, as PTCs have been strongly associated with potential pathogenic mechanisms for congenital supravalvar aortic stenosis (16, 18, 60, 61). The variant was classified as pathogenic according to the American College of Medical Genetics and Genomics and the Association for Molecular Pathology (ACMG/AMP) criteria (PS4, PVS1, PM2) (62). This classification indicates that the c.1621C>T variant has been identified in affected individuals relative to controls, that PTCs in *ELN* are a known mechanism of disease, and that the allele frequency is extremely low in population databases (16, 60, 61, 63).

The proband case also carries a pathogenic *BRAF* variant. *BRAF* encodes a central serine/threonine kinase in the RAS/ mitogen-activated protein kinase (RAS/MAPK) signaling pathway (23, 24, 64). The RAS/MAPK pathway is critical for embryogenesis and cardiac morphogenesis, and its dysregulation has been implicated in the RASopathies, a group of developmental syndromes with frequent cardiac involvement (23–25, 65, 66). Gain-of-function variants in the *BRAF* gene can potentially lead to aberrant RAS/MAPK activation, resulting in dysregulated cell proliferation and differentiation, and ultimately to structural abnormalities of the pulmonary valve (25, 67). Notably, Sun et al. (2022) reported that patients carrying *BRAF* variants were diagnosed with congenital valvar pulmonary stenosis, with or without hypertrophic cardiomyopathy, and had a poorer prognosis compared to those without structural cardiac defects (25).

Moreover, additional clinical review identified craniofacial features, including hypertelorism, broad forehead, coarse facial appearance, and prognathism. Together with congenital pulmonary valvar stenosis and the pathogenic *BRAF* c.1460T>G; p.(Val487Gly) variant, these findings may raise the possibility of a syndromic phenotype within the RASopathy spectrum, potentially a BRAF-related cardio-facio-cutaneous/Noonan-like presentation. This interpretation is biologically concordant given the known role of *BRAF* in the RAS/MAPK pathway and the association of RASopathies with pulmonary valve involvement (22, 23). However, no functional analysis was performed in this case, and the specific effect of this variant on RAS/MAPK signaling in this individual was not assessed. In addition, formal dysmorphological evaluation was not available. Therefore, the potential relationship between the BRAF variant, the craniofacial findings, and the pulmonary valve phenotype should be interpreted cautiously as clinically suggestive and rather than diagnostic.

Another variant identified in the proband is the *NOTCH1* p. Leu2429Arg. The NOTCH1 receptor activates the Notch signaling pathway, which plays a pivotal role in developmental processes, including primordial valve formation, cardiac embryogenesis, and vasculogenesis (68). Pathogenic *NOTCH1* variants have been associated with congenital cardiac phenotypes, particularly aortic valve disease (68, 69). In the present case, the variant has been cataloged as VUS based on criteria PM2 and PP2 (60, 62). PM2 was applied because of its very low population frequency, whereas PP2 was considered in the context of prior gene-level evidence supporting intolerance of *NOTCH1* to missense variation, including a pLI of 1 and a missense Z-score of 5.16 (60, 70). However, these criteria are insufficient to establish pathogenicity. Therefore, the potential role of this *NOTCH1* variant as a phenotypic modifier should be regarded only as a hypothesis. Moreover, modifier effects are difficult to demonstrate even in large cohorts, and they cannot be inferred from a single case without segregation analysis, functional validation, and additional genotype–phenotype evidence (71, 72). Accordingly, the clinical relevance of this *NOTCH1* VUS remains uncertain.

These findings raise the possibility of a multilocus genetic context in which more than one clinically relevant variant may coexist in a patient with complex CHD. Nevertheless, this case does not establish gene–gene interaction or prove that all identified variants contributed directly to the phenotype. Rather, it supports the need for cautious genotype–phenotype interpretation, family-based studies, and functional validation when evaluating complex CHD presentations. Clinical variability among genetic cardiomyopathies is well documented, suggesting that gene–gene interactions and environmental factors may further modulate phenotypic expression (73).

Disparities in cardiovascular health have been extensively reported, disproportionately affecting racial and ethnic minorities and socioeconomically disadvantaged populations (74–76). In Ecuador, human genetics research remains limited, and cardiogenetics is an even less developed field (7–10, 38, 42–44, 73, 77, 78). Patients with CHDs from this region are consequently likely underrepresented in the scientific literature and in international genomic databases. Case reports such as this one are therefore important not only for documenting rare clinical presentations, but also for building local evidence, expanding access to molecular diagnostics, and contributing data from historically underrepresented populations.

Furthermore, the patient showed an admixed genetic background with European, Native American, and African ancestry components, with European ancestry being the predominant contribution, followed by Native American and African ancestry. This finding reflects the complex demographic history of Ecuador and highlights the importance of considering population structure in genomic medicine (7–10, 38, 42–44, 73, 77–79). Greater representation of admixed Latin American populations is essential to improve variant interpretation, reduce diagnostic uncertainty, and advance equitable precision medicine.

The limitations of the study include that because this is a single pediatric case without functional validation, segregation analysis, or informative family history, the study cannot establish causality, quantify the contribution of each variant, or confirm a multilocus mechanism. The genotype–phenotype interpretation should therefore be regarded as biologically plausible. Another limitation is that environmental, maternal, and epigenetic developmental factors were not evaluated; therefore, the observed phenotype should not be attributed exclusively to the identified genetic variants. However, it describes an unusual combination of coexisting congenital obstructive cardiac lesions and integrates clinical findings with molecular data, highlighting the role of genomics in CHD diagnostics. The inclusion of ancestry analysis provides broader genomic context and adds evidence from an underrepresented Latin American population.

In summary, this case illustrates how complex CHD may arise from the interaction of multiple molecular mechanisms in the context of a diverse ancestral background. Genomic evaluation can provide insights to advance cardiogenetic research in Ecuador, with direct implications for personalized healthcare. The patient's parents expressed gratitude for the study and for the opportunity to better understand the genetic basis of their child's condition.

## Data Availability

The data presented in the study are deposited in the Sequence Read Archive from National Center of Biotechnology Information, accession number PRJNA1484652.
